# Novel control of lactate dehydrogenase from the freeze tolerant wood frog: role of posttranslational modifications

**DOI:** 10.7717/peerj.12

**Published:** 2013-02-12

**Authors:** Jean Abboud, Kenneth B. Storey

**Affiliations:** Institute of Biochemistry, Department of Biology and Department of Chemistry, Carleton University, Canada

**Keywords:** *Rana sylvatica*, Muscle glycolysis, Freeze tolerance, Enzyme regulation, Posttranslational modification, Dehydration, Cryobiology

## Abstract

Lactate dehydrogenase (LDH), the terminal enzyme of anaerobic glycolysis, plays a crucial role both in sustaining glycolytic ATP production under oxygen-limiting conditions and in facilitating the catabolism of accumulated lactate when stress conditions are relieved. In this study, the effects on LDH of in vivo freezing and dehydration stresses (both of which impose hypoxia/anoxia stress on tissues) were examined in skeletal muscle of the freeze-tolerant wood frog, *Rana sylvatica*. LDH from muscle of control, frozen and dehydrated wood frogs was purified to homogeneity in a two-step process. The kinetic properties and stability of purified LDH were analyzed, revealing no significant differences in *V*_max_, *K*_*m*_ and *I*_50_ values between control and frozen LDH. However, control and dehydrated LDH differed significantly in *K*_*m*_ values for pyruvate, lactate, and NAD, *I*_50_ urea, and in temperature, glucose, and urea effects on these parameters. The possibility that posttranslational modification of LDH was responsible for the stable differences in enzyme behavior between control and dehydrated states was assessed using ProQ diamond staining to detect phosphorylation and immunoblotting to detect acetylation, methylation, ubiquitination, SUMOylation and nitrosylation of the enzyme. LDH from muscle of dehydrated wood frogs showed significantly lower levels of acetylation, providing one of the first demonstrations of a potential role for protein acetylation in the stress-responsive control of a metabolic enzyme.

## Introduction

The wood frog, *Rana sylvatica*, is one of a very few vertebrate animals that can endure long-term whole body freezing (Storey & Storey, 1988) and the only one in its family (*Ranidae*) known to tolerate more than 10 h of freezing (Voituron et al., 2009). Wood frogs spend the winter under leaf litter in moist parts of the forest floor; once covered by an insulating blanket of snow, they are sheltered from the harshest winter conditions but temperature in their microenvironment can still drop several degrees below the freezing point of their body fluids. The frogs can experience multiple freeze-thaw episodes over the winter and can endure the conversion of 65%–70% of total body water into ice (Storey & Storey, 1988; Storey & Storey, 2004). A key adaptation for freezing survival is the production of glucose as a cryoprotectant that is synthesized mainly from liver glycogen reserves and distributed to other organs via the blood during the early minutes/hours of freezing (Storey, 1987; Storey & Storey, 1986). Well-developed tolerances of cellular dehydration and anoxia/ischemia by this species are also integral to freezing survival (Churchill & Storey, 1993; Holden & Storey, 1997). Indeed, once breathing and circulation are halted by freezing, frogs must rely on anaerobic glycolysis for their ATP needs for days or weeks until thawing occurs. This is evidenced by the accumulation of lactate in organs over time during freezing; for example, in skeletal muscle, lactate rose from 2 µmol/g wet weight (gww) in control frogs to 15 µmol/gww after 4 d frozen at −4 °C (Storey & Storey, 1984; Storey & Storey, 1986).

The importance of anaerobic glycolysis to cell, organ and whole animal survival during freezing led us to question whether the enzyme lactate dehydrogenase (LDH) was modified or regulated in any way that would support its role in freezing survival. LDH (EC 1.1.1.27) has been the subject of many studies because of its crucial role in both the production and catabolism of lactate via the reversible reaction:

\begin{eqnarray*} \displaystyle \mathrm{pyruvate}+\mathrm{NADH}+{\mathrm{H}}^{+}\leftrightarrow \mathrm{lactate}+\mathrm{NAD}.&&\displaystyle \end{eqnarray*}

Our particular interest in the present study was the potential role of reversible posttranslational modification (PTM) of LDH as a mechanism influencing enzyme function/properties in response to the stresses of freezing and dehydration. PTMs can have strong effects on enzymes including altering activity, kinetic parameters, protein stability, protein–protein or subunit–subunit interactions, and subcellular localization (Cohen, 2002). Reversible phosphorylation has long been known to have major regulatory effects on many enzymes of intermediary metabolism but many other PTMs also occur including acetylation, methylation, ubiquitination, SUMOylation, and others. The consequences of these other PTMs for enzyme regulation in comparative animal systems is only beginning to be investigated. Indeed, our lab recently demonstrated that LDH from skeletal muscle of an anoxia tolerant turtle, *Trachemys scripta elegans*, was both phosphorylated and acetylated in response to anoxia (Xiong & Storey, 2012). The present study analyzes purified LDH from wood frog skeletal muscle, evaluating the effects of whole animal freezing and dehydration exposures on LDH properties and assessing the potential involvement of eight different PTMs in contributing to stress-responsive LDH control.

## Material and methods

### Chemicals and animals

Chemicals were purchased from Sigma Chemical Company (St. Louis, MO) or BioShop (Burlington, ON). Male wood frogs (*R. sylvatica*; body mass 4–7 g) were captured from spring breeding ponds in the Ottawa area. Animals were washed in a tetracycline bath and held in plastic containers with damp sphagnum moss at 5 °C for two weeks prior to experimentation. Control frogs were sampled from this condition. For freezing exposure, frogs were placed in closed plastic boxes with damp paper toweling on the bottom, and put in an incubator set at −4 °C. Animals were held at this temperature for 45 min which is sufficient time for body temperature to chill below 0.5 °C (the equilibrium freezing point of wood frog body fluids) and trigger nucleation. Subsequently, incubator temperature was adjusted upwards to −3 °C and the timing of a 24 h freezing exposure began. For dehydration, frogs were individually weighed and tagged. Frogs were placed in large desiccators that contained silica gel desiccant on the bottom, separated from the frogs by a 1 cm thickness of sponge. Desiccators were held at 5 °C and the frogs were quickly removed and weighed at intervals. Frogs lost total body water at a mean rate of ∼0.5% per hour and were sampled when total body water loss rose to ∼40%. Control and experimental frogs were sacrificed by pithing, followed by rapid dissection, and freezing of tissue samples in liquid nitrogen. Tissue samples were stored at −80 °C until use. Conditions for animal care, experimentation, and euthanasia were approved by the Carleton University Animal Care Committee (B09-22) in accordance with guidelines set down by the Canadian Council on Animal Care.

### Preparation of tissue extracts and enzyme purification

Skeletal muscle (from thigh) samples were quickly weighed and mixed 1:10 w:v in ice-cold buffer A (50 mM Tris, pH 8.0, 10% v/v glycerol, 5 mM 2-mercaptoethanol, 3 mM EDTA, 3 mM EGTA, 20 mM β-glycerophosphate) that contained protein kinase and protein phosphatase inhibitors; after the quick addition of a few crystals of phenylmethylsulfonyl fluoride (PMSF), the sample was immediately homogenized on ice with a Diamed Pro 200 homogenizer. Homogenates were then centrifuged at 10,000 rpm for 30 min at 4 °C and the supernatant was stored on ice. A 1 mL aliquot of supernatant was then diluted 1:2 v:v in buffer A and loaded onto a 5×1 cm column of Cibacron blue (bead bed at 7 mm from bottom) equilibrated in buffer A, washed to remove unbound protein and eluted with 40 mL of buffer A containing 0.4 M KCl, 3 mM pyruvate and 3 mM NADH. The fractions with highest activity were pooled and diluted 1:5 v:v in buffer A. The diluted sample was then applied to a second Cibacron blue column equilibrated as above and eluted with a gradient of 0–2 M KCl in buffer A. Purified LDH was collected for enzyme assay and a portion was further concentrated for analysis by SDS-polyacrylamide gel electrophoresis (PAGE). SDS-PAGE and silver staining were carried out as in Xiong & Storey (2012).

### Enzyme assays

LDH activity was measured by monitoring the consumption or production of NADH at 340 nm using a Multiskan Spectrum microplate reader. Optimal assay conditions for the forward reaction were determined to be 50 mM Tris pH 7.0, 0.15 mM NADH, and 2 mM pyruvate in a 200 µL total volume; assays were typically started by the addition of 10 µL of purified enzyme. Optimal assay conditions for the reverse reaction were 50 mM Tris pH 8.0, 1 mM NAD, and 22.5 mM L-lactate. Routine assays were run at room temperature (22 °C). *K*_*m*_ values and *I*_50_ pyruvate were determined at constant, saturating co-substrate concentrations, as above. *I*_50_ for urea or hydrogen peroxide were determined at constant, subsaturating levels of substrate. For analysis of the pH dependence of LDH activity, assays were conducted at 22 °C under optimal substrate concentrations in different 50 mM buffers: MES (pH 5–6.5), Tris (pH 7–9), or CAPS (pH 9.5–10.5).

Soluble protein concentrations were determined using the Coomassie blue G-250 dye-binding method with the Bio-Rad Laboratories prepared reagent and bovine serum albumin as the standard.

### Arrhenius plots

LDH was assayed under *V*_max_ substrate conditions over a temperature range from 5–36 °C. Activation energy (*E*_*a*_) was determined in kJ/mol from the Arrhenius equation. For low temperatures (<20 °C), the Multiskan Spectrum microplate reader was equilibrated to the appropriate temperature in a Precision 815 low temperature incubator (Thermo Scientific, Ottawa, ON) whereas for high temperatures (>20 °C), an Echotherm heating plate (Torrey Pines Scientific, USA) was used and the temperature was held constant in the microplate reader. In all cases, filled microplates were temperature-equilibrated for ∼10 min before reactions were initiated by addition of NADH/NAD (also temperature-equilibrated). Temperature was confirmed both before and after assay by a thermistor placed in a microplate well.

### Differential scanning fluorimetry

Pure LDH (concentration greater than 0.01 µg/µL), DSF buffer (100 mM potassium phosphate, pH 7.0, 150 mM NaCl), any additional reagents and 40× diluted SYPRO orange dye (Invitrogen) were mixed in a 20 µL final volume in the wells of thin-walled PCR plates. Plates were sealed and placed in a Bio-Rad iQ5 PCR instrument. SYPRO orange fluorescence was measured (excitation filter: 490±20 nm, emission filter: 625±30 nm) during monitoring over a temperature gradient from 15 °C to 93 °C (1 °C increments with 30 s reads). Analysis of fluorescence intensity using OriginPro 8.5 and the Boltzmann distribution curve was used to calculate the midpoint temperature of the protein-unfolding transition, known as the protein melting temperature (*T*_*m*_) (Biggar, Dawson & Storey, 2012).

### Western blotting

Samples of purified LDH were concentrated 7-fold in 2 mL Centricons (Millipore Bioscience) by centrifugation at 7500 g for 10 min. Concentrated purified enzyme was then mixed 2:1 v:v with SDS loading buffer (100 mM Tris buffer, pH 6.8, 4% w:v SDS, 20% v:v glycerol, 0.2% w:v bromophenol blue) with 10% v:v 2-mercaptoethanol added fresh and then boiled for 5 min, cooled on ice and frozen at −20 °C. Aliquots (20 µL) of thawed, well-mixed sample were added into the wells of 10% SDS-PAGE gels. Gels were run at 180 V for 45 min in running buffer containing 25 mM Tris-base, 250 mM glycine and 0.1% w:v SDS. Proteins were wet-transferred to PVDF membranes using a current of 160 mA for 1.5 h at 4 °C and the Bio-Rad Mini Trans-Blot Cell apparatus. Transfer buffer contained 25 mM Tris-base (pH 8.8), 192 mM glycine, and 20% v:v methanol, chilled at 4 °C. Membranes were then incubated with antibody overnight at 4 °C. All antibodies used in this study were made in rabbits and diluted 1:1000 v:v in TBST (20 mM Tris base, pH 7.6, 140 mM NaCl, 0.05% v/v Tween-20) before use: anti-acetyl (Santa Cruz Biotechnology; cat. # sc 8663-R,), anti-methyl arginine (Covalab; cat. # mab0002-0), anti-methyl lysine (Biosciences Inc.; cat. # SPC-158F), anti-SUMO 1 and 2/3 (generous gift from Dr. JM Hallenbeck, NINDS, NIH, Bethesda, MD), anti-ubiquitin (Abcam; cat. # ab19247), and anti-nitrosyl (Abcam; cat. # ab50185). Unbound primary antibody was removed with three 5 min washes with TBST and the membrane was incubated with HRP-conjugated anti-rabbit secondary antibody (BioShop, diluted 1:4000 v:v in TBST) for 30 min at room temperature, followed by three 5 min washes with TBST. Membranes were then developed using Western Lighting Chemiluminescence Plus reagents (NEN, Perkin Elmer) following manufacturer’s protocols, washed three times for 5 min and signal was detected using enzymatic chemiluminescence (ECL). Detection used the ChemiGenius Bioimaging System (Syngene, MD) and band densities were quantified using GeneTools software (v3.00.02). Genedirex 10.5–175 kDa protein ladders were run in selected lanes to assess the subunit molecular mass of LDH. Subsequently, gels were re-stained for 5 min with Coomassie blue (25% w/v Coomassie Brilliant Blue R in 50% v/v methanol, 7.5% v/v acetic acid) and destained for 10 min with destaining mix (50% v/v methanol, 10% v/v acetic acid in distilled deionized H_2_O [ddH_2_O]). LDH band intensities were normalized against the corresponding Coomassie blue stained band to correct for any variations in sample loading.

### ProQ diamond phosphoprotein staining

Purified enzyme was mixed 1:1 v:v with SDS loading buffer and prepared for electrophoresis as above. Aliquots of 30 µL were added to the wells of 10% SDS-PAGE gels and electrophoresis was carried out as above. Gels were washed twice in fixing solution (50% v:v methanol, 10% v:v acetic acid in ddH_2_O) and left overnight in this solution at ∼22 °C. The following day, the gel was washed three times with ddH_2_O for 10 min each and then stained with ProQ Diamond phosphoprotein stain (Invitrogen, Eugene, OR) for 90 min. During staining the gel container was covered with tin foil to exclude light; the gel also remained covered for the next steps. The gel was washed three times with ddH_2_O for 10 min each, destained by washing twice for 30 min each with ProQ Diamond destaining solution (20% v:v acetonitrile, 50 mM sodium acetate, pH 4) and then washed again with ddH_2_O as above. Fluorescent bands on the gel were visualized with the ChemiGenius and band intensities were quantified. Subsequently, gels were restained with Coomassie blue as described above.

### Dot blots

Dot blots were used to analyze and identify protein posttranslational modifications using the method of Dawson, Bell & Storey (in press). Purified LDH (7-fold concentrated as described above) was applied to nitrocellulose membranes using a Bio-Dot^®^ microfiltration apparatus that was pre-washed twice with TBS (20 mM Tris base, 140 mM NaCl, pH 7.6). In brief, samples passed through the apparatus by gravity and were then washed twice with TBST. The membrane was separated with a razor blade for respective antibody probing, followed by three 5 min washes with TBST, blocked with 1 mg/ml poly(vinyl alcohol) in TBST for 30 s, and then washed again with TBST as above followed by probing and visualization as described for western blotting.

### Data and statistical analysis

A Microplate Analysis (MPA) Program was used to analyze enzyme rates and kinetic parameters were derived using a nonlinear least squares regression computer program, Kinetics 3.51 (Brooks, 1992). IBM SPSS-14 Statistics Desktop v21.0.0 software was used for all univariate and error plot analysis. As appropriate, statistical analysis used either the Student’s independent *t*-test or a one-way or two-way ANOVA linked with the Bonferroni or Tukey’s post-hoc test (Ennos, 2006). Values for “*n*” denote the number of independent preparations of enzyme tested, each preparation being derived from pooled muscle samples from at least 2 frogs.

## Results

### Purification of wood frog muscle LDH

**Figure 1:**
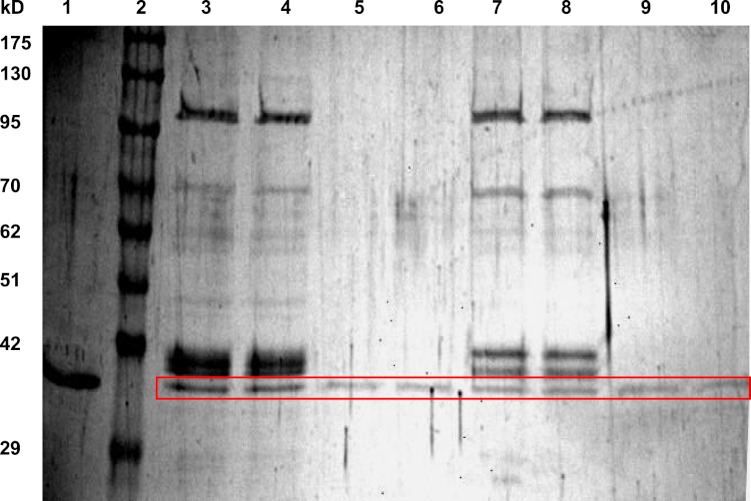
SDS-PAGE gel showing purification of LDH from wood frog hind leg skeletal muscle. Lanes are: (1) purified turtle muscle LDH (Xiong & Storey, 2012), (2) Genedirex 10.5–175 kD protein ladders, (3, 4) first Cibacron blue column for LDH from control muscle, (5, 6) second Cibacron blue column for LDH from control muscle, (7, 8) first Cibacron blue column for LDH from frozen muscle, and (9, 10) second Cibacron blue column for LDH from frozen muscle. The gel was stained with silver.

LDH from hind leg skeletal muscle was purified from control (5 °C acclimated), frozen (24 h frozen at −3 °C) and dehydrated (40% total body water loss) wood frogs using a two-step method. Table 1 shows a typical purification for LDH from muscle of 24 h frozen wood frogs and Fig. 1 shows a representative SDS-PAGE gel for LDH purification from muscle of control and frozen frogs. The first purification step was a Cibacron blue affinity column with elution of LDH using a mixture of pyruvate + NADH. The second step was elution off a second Cibacron blue affinity column using a KCl gradient. The first Cibacron blue column gave a 1.43-fold purification and a yield of 52.2% whereas the second column gave a 26.5-fold purification and a 41.6% yield. SDS-PAGE analysis showed single bands of LDH protein for all purified preparations; Fig. 1 shows examples for LDH purification from control and frozen frogs. The calculated subunit molecular mass of wood frog muscle LDH was 34.4±0.7 kD, very close to the value for purified turtle muscle LDH seen in lane 1 (36.9 kD) (Xiong & Storey, 2012).

**Table 1:** Purification of LDH from skeletal muscle of freeze-exposed *R. sylvatica*.

Step	Total protein (mg)	Total activity (Units)	Yield (%)	Fold purification	Specific activity (U/mg)
Crude	1.93	2.37	N/A	n/a	1.2
Cibacron blue 1	0.70	1.24	52	1.43	1.8
Cibacron blue 2	0.03	0.99	42	26.5	32.6

**Notes.**

Activities were measured in the reverse direction.

### Activities and kinetic analysis of purified LDH from control, frozen and dehydrated muscle

The activity of purified wood frog muscle LDH in the pyruvate reductase forward direction was approximately three times higher than the rate of the lactate-oxidizing reverse reaction for the enzyme from control, 24 h frozen or 40% dehydrated frogs (hereafter designated as control LDH, frozen LDH and dehydrated LDH). In the forward, pyruvate-utilizing, direction the pH optimum of LDH was in the pH 6–8 range for LDH for all three animal states whereas in the lactate-oxidizing direction activity was optimal and nearly constant between pH 8.0–10.5.

### Kinetic parameters of the LDH reverse reaction

Table 2 shows kinetic parameters for muscle LDH purified from control, frozen and dehydrated frogs. There was no significant difference in the *V*_max_ of purified LDH in the reverse reaction (31.6–35.5 U/mg) between the three animal states when assayed under optimal assay conditions at pH 8 and 22 °C. Similarly, Michaelis constants (*K*_*m*_) for lactate and NAD did not differ between control and frozen states but the *K*_*m*_ values for these substrates were about 50% lower and significantly different for LDH purified from muscle of dehydrated frogs (*p* < 0.01,*n* = 10). Inhibition of LDH by urea and hydrogen peroxide was also tested. Values for *I*_50_ (concentration that reduces activity by 50%) in the reverse direction were not significantly different between control and frozen LDH and were ∼3.1 M for urea and 548–613 mM for H_2_O_2_ , respectively. However, *I*_50_ urea was significantly lower for LDH from dehydrated frogs (2.12 M), 35% lower than the control value (*p* < 0.01,*n* = 4). Activation of LDH by fructose-1,6-bisphosphate (FBP), as reported by some authors (Brown & Wittenberger, 1972; Eisenberg, Elchisak & Rudd, 1976), was also tested but no activation was found when either control or frozen LDH was analyzed in the presence of up to 3.25 mM FBP in either the forward or the reverse direction.

**Table 2:** Kinetic parameters of purified muscle LDH from control, frozen and dehydrated *R. sylvatica*.

Enzyme parameter	Control	Frozen	Dehydrated
*Reverse reaction (lactate* → *pyruvate)*			
*V*_max_ at 22 °C (U/mg)	35.52±0.07	32.55±0.44	31.56±0.17
*K*_*m*_ lactate, pH 8, 22 °C (mM)	11.02±0.64	11.99±0.85	5.60±0.50^a^
*K*_*m*_ NAD, pH 8, 22 °C (mM)	1.38±0.05	1.40±0.04	0.64±0.04^a^
*I*_50_ urea, pH 8, 22 °C (M)	3.16±0.12	3.10±0.10	2.12±0.03^a^
*I*_50_H_2_O_2_ , pH 8, 22 °C (mM)	548±3	613±1	N/A
*Forward reaction (pyruvate* → *lactate)*			
*K*_*m*_ pyruvate, pH 7, 22 °C (mM)	0.16±0.01	0.14±0.01	0.13±0.01^c^
*K*_*m*_ pyruvate, pH 7, 9 °C (mM)	0.09±0.01^b^	N/A	0.085±0.007^b^
*K*_*m*_ pyruvate, pH 7, 32 °C (mM)	0.26±0.004^b^	N/A	0.195±0.002^b^ ^,^ ^c^
*I*_50_ pyruvate, pH 7, 22 °C (mM)	7.93±0.34	9.73±1.17	8.26±0.76

**Notes.**

Data are means ±SEM, *n* = 4 samples from different frogs for most parameters and *n* = 10 for *K*_*m*_ values at 22 °C. N/A, not available. Statistical testing used ANOVA followed by a Bonferroni post-hoc test.

a Significantly different from the corresponding control value (one-way ANOVA), *p* < 0.01

b Significantly different from the corresponding value at 22 °C (two-way ANOVA), *p* < 0.01

c Significantly different from the corresponding control value at the same temperature (two-way ANOVA), *p* < 0.01.

**Figure 2:**
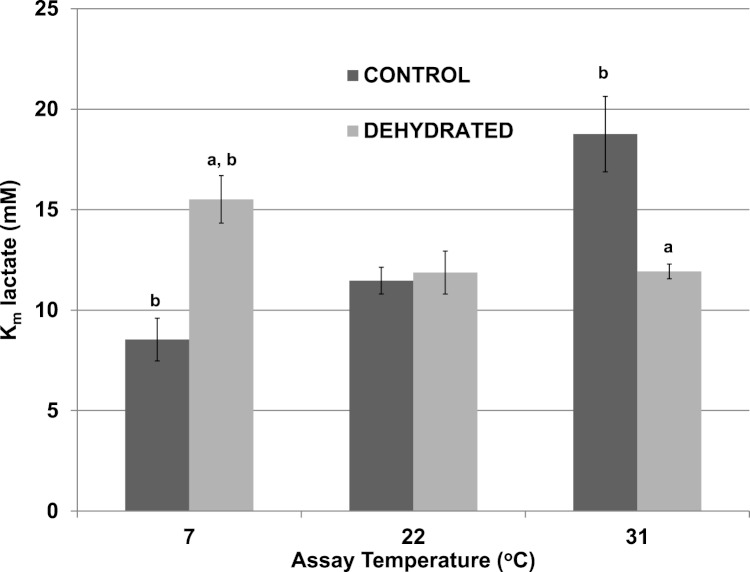
Effect of temperature on the *K*_*m*_ lactate of purified wood frog muscle LDH in the presence of 400 mM glucose, comparing LDH from control and dehydrated frogs. Assays were at 7 °C, 22 °C and 31 °C, all in the presence of 400 mM glucose and at pH 8. Data are means ± SEM, *n* = 4 independent determinations. a – significantly different from the value for control LDH assayed at the same temperature using two-way ANOVA and Bonferroni Post Hoc test, *p* < 0.05; b – significantly different from the same animal state assayed at 22 °C (pH 8) using two-way ANOVA, *p* < 0.05.

The effects of temperature change (7 °C, 22 °C, 31 °C) on the *K*_*m*_ lactate were evaluated for purified control and dehydrated LDH in the presence of 400 mM glucose, the sugar that accumulates as a protectant in the tissues of both frozen and dehydrated frogs. At 22 °C, glucose at 400 mM had no effect on the *K*_*m*_ lactate of control LDH (11.47±1.06 mM) (Fig. 2), as compared with the value without glucose seen in Table 2. However, the presence of 400 mM glucose increased the *K*_*m*_ lactate of dehydrated LDH to 11.87±1.19 mM (*p* < 0.05,*n* = 4) (Fig. 2), about twice the value without glucose (Table 2) but this did not significantly differ from the control *K*_*m*_ value in either the presence or absence of glucose. At 7 °C in the presence of 400 mM glucose the *K*_*m*_ lactate of control LDH significantly decreased to 8.53±0.66 mM (*p* < 0.05,*n* = 4) whereas that of dehydrated LDH (15.51±1.07 mM) significantly increased from the corresponding value at 22 °C (*p* < 0.05,*n* = 4) and was also significantly higher than the control value at 7 °C (*p* < 0.05,*n* = 4). At a warmer temperature (31 °C) in the presence of 400 mM glucose the *K*_*m*_ lactate of control LDH increased by over 60% to 18.76±1.88 mM (*p* < 0.05,*n* = 4) whereas the *K*_*m*_ of dehydrated LDH was unchanged (11.93±0.36 mM), as compared to values at 22 °C.

**Figure 3:**
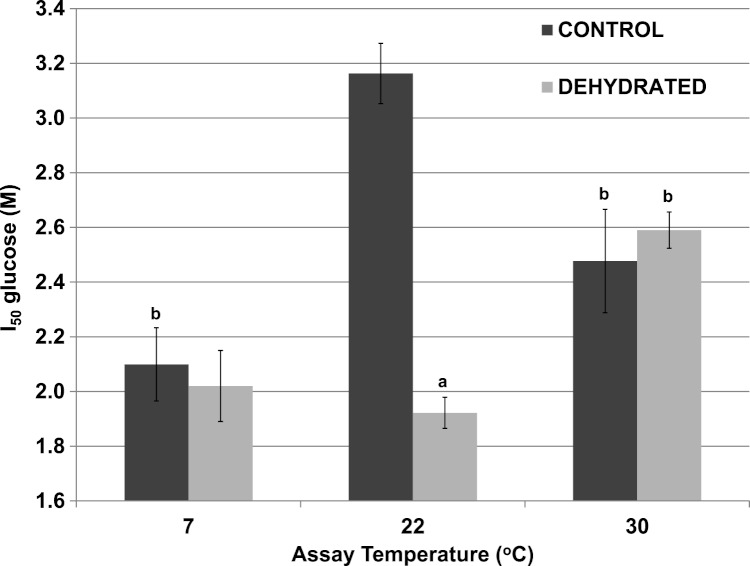
Effect of temperature on the *I*_50_ glucose for the reverse reaction of purified wood frog muscle LDH, assayed at 7 °C, 22 °C, and 30 °C. Data are means ±SEM, *n* = 4 independent determinations. a – significantly different from the value for control LDH assayed at the same temperature using two-way ANOVA and Bonferroni Post Hoc test, *p* < 0.05; b – significantly different from the same animal state assayed at 22 °C (pH 8) using two-way ANOVA, *p* < 0.01.

Although 400 mM glucose had differential effects on the *K*_*m*_ lactate from muscle of control and dehydrated wood frogs, higher glucose was inhibitory for both enzymes. Figure 3 shows *I*_50_ values for glucose for control and dehydrated LDH assayed in the reverse, lactate-utilizing direction. *I*_50_ glucose at 22 °C for control LDH was 3.16±0.13 M whereas the value for dehydrated LDH was 1.92±0.13 M, 60% lower and significantly different from the control value (*p* < 0.05,*n* = 4). However, at a lower temperature (7 °C), the *I*_50_ glucose for control LDH decreased significantly (2.10±0.11 M) as compared with the value at 22 °C (*p* < 0.01,*n* = 4) and was not different from that of dehydrated LDH (2.02±0.06 M). At a higher temperature (30 °C), the *I*_50_ glucose values of control (2.48±0.19 M) and dehydrated (2.59±0.07 M) LDH were not different from each other but the value for control LDH was significantly lower than its corresponding value at 22 °C (*p* < 0.01,*n* = 4) and dehydrated LDH was significantly higher (*p* < 0.01,*n* = 4).

**Figure 4:**
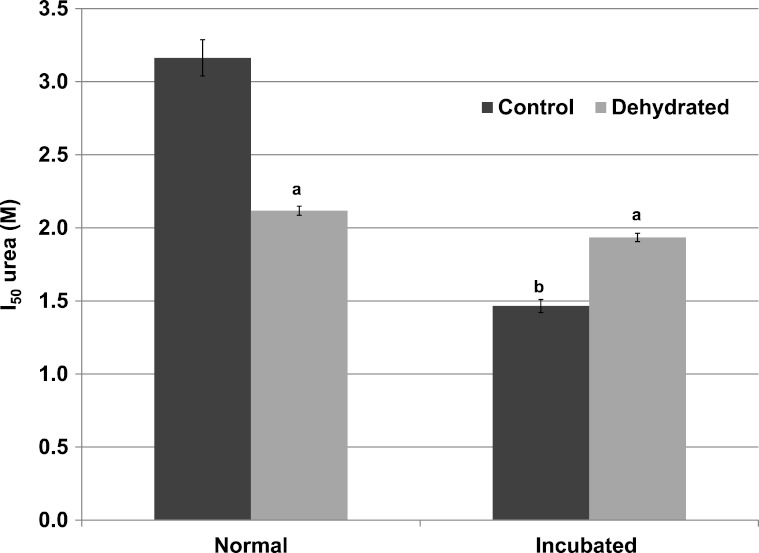
Effect of urea on the reverse reaction of purified wood frog muscle LDH: *I*_50_ urea values for enzyme exposed immediately to urea (normal) versus given a 35 min pre-incubation period with urea (incubated). Data are means ± SEM, *n* = 4 independent determinations. a – significantly different from control LDH under the same assay conditions using two-way ANOVA and Bonferroni Post Hoc test, *p* < 0.01; b – significantly different from the same animal state under normal assay conditions (with urea but no incubation period, room temperature, pH 8) using two-way ANOVA, *p* < 0.01.

Inhibitory effects of urea on LDH were different when assessed during immediate exposure of the enzyme to urea as opposed to after a 35 min pre-incubation of the enzyme with urea before the reaction was initiated. When all reagents including urea were present before starting the reaction by LDH addition, the *I*_50_ urea of dehydrated LDH (2.12±0.03 M) for the reverse lactate-oxidizing reaction was approximately two-thirds of the value for control LDH (3.16±0.12 M) (*p* < 0.01,*n* = 4) (Fig. 4). However, when the enzyme was pre-incubated for 35 min with urea before the reaction was started, the *I*_50_ urea of control LDH was significantly reduced by more than 50% to 1.47±0.04 M (*p* < 0.01,*n* = 4) whereas that of dehydrated LDH (1.93±0.03 M) was unchanged.

**Figure 5:**
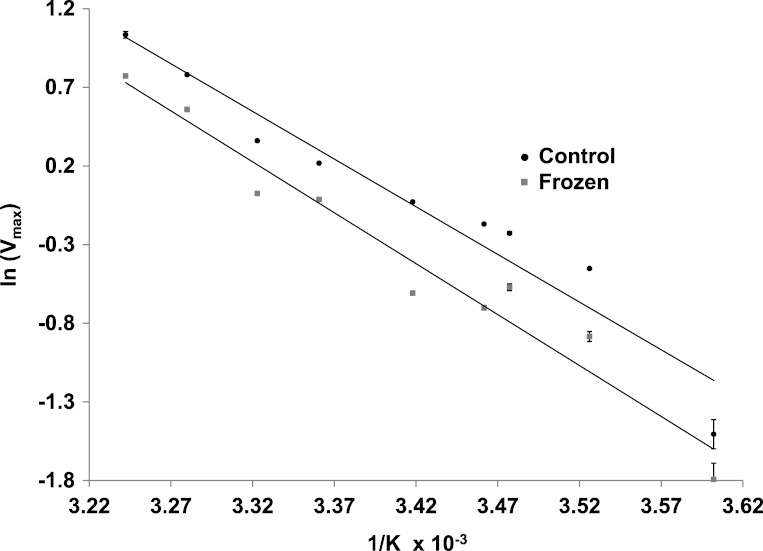
Arrhenius plots for the reverse reaction for LDH purified from muscle of control and frozen frogs. Maximal activities were measured at nine temperatures from 5–36 °C. Data are means ± SEM, *n* = 3 independent determinations.

The effects of temperature on the maximal activity of the reverse direction of wood frog muscle LDH were measured at nine temperatures over the range 5–36 °C and evaluated using Arrhenius plots (Fig. 5). Plots were linear over the full temperature range tested for LDH purified from muscle of both control and frozen frogs. Calculated activation energies (*E*_*a*_) were not statistically different for control (50.4±2.2 kJ/mol) and frozen (53.9±2.6 kJ/mol) LDH.

### Kinetic parameters for the LDH forward reaction

Kinetic parameters for the forward reaction were also measured for purified control, frozen and dehydrated LDH. The *K*_*m*_ pyruvate did not differ between control and frozen LDH but the *K*_*m*_ pyruvate of dehydrated LDH was 20% lower and significantly different than the control value (*p* < 0.01,*n* = 10) (Table 2). The *I*_50_ pyruvate was 7.9–9.7 mM and not significantly different between the three metabolic states. Table 2 also shows that temperature had an effect on *K*_*m*_ pyruvate. The *K*_*m*_ pyruvate was positively correlated with temperature for both control and dehydrated LDH (*p* < 0.01,*n* = 4) and increased by 2–3 fold between 7 °C and 32 °C.

### Structural stability of LDH

**Figure 6:**
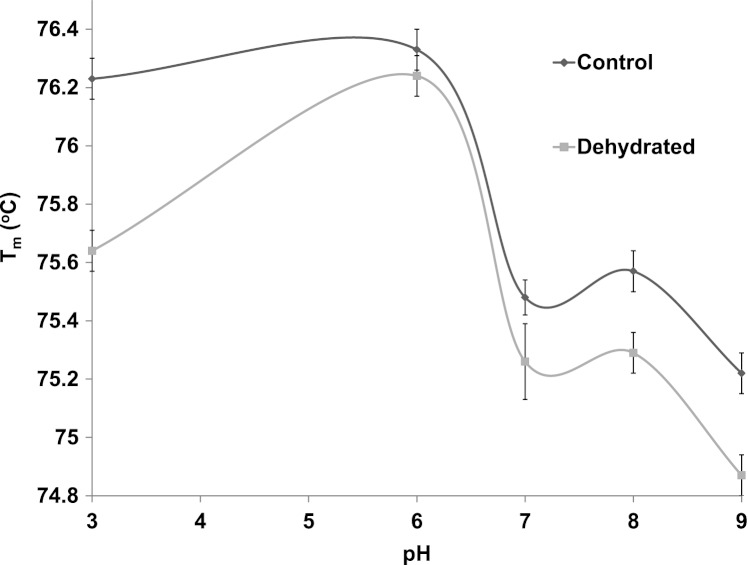
The effect of pH change on the melting temperature (*T*_*m*_) of purified LDH from muscle of control and dehydrated wood frogs as determined by differential scanning fluorimetry.

Differential scanning fluorimetry (DSF) is an efficient method that assesses the thermal unfolding of proteins in the presence of a fluorescent dye (Niesen, Berglund & Vedadi, 2007). Heat is a denaturant to all enzymes and causes their hydrophobic core to become exposed allowing a hydrophobic dye to bind within these regions and resulting in the emission of light. DSF was used to test the stability of purified control and dehydrated LDH against heat in the presence of different supportive versus disruptive conditions. The *T*_*m*_ value, the temperature that results in 50% unfolding, of LDH was measured in the presence versus absence of stabilizers such as KCl and destabilizers such as urea. *T*_*m*_ values at 22 °C and pH 6 were very similar for control and dehydrated LDH (both approx. 76.2 °C) but diverged at both lower and higher pH values (Fig. 6). *T*_*m*_ values were negatively correlated with pH within the range from pH 6–9 for both control and dehydrated LDH. Differences between control and dehydrated LDH were most pronounced for the enzyme exposed to pH 3 where *T*_*m*_ values differed by 0.6 °C as the result of a decrease in *T*_*m*_ for dehydrated LDH at the acid pH value.

**Figure 7:**
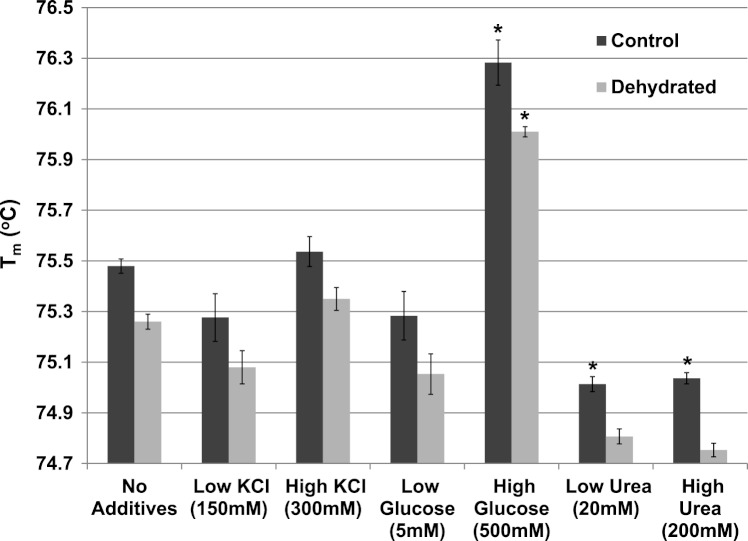
The effect of various additives (KCl, glucose, urea) on the *T*_*m*_ of purified LDH from muscle of control and dehydrated wood frogs. Data are means ±SEM, *n* = 3 independent determinations. * – significantly different from the same animal state without additives using one-way ANOVA and Tukey’s Post Hoc test, *p* < 0.05.

The effect of various additives (KCl, glucose, urea) on *T*_*m*_ were also evaluated for both control and dehydrated LDH at pH 7 (Fig. 7). Neither low (150 mM) nor higher (300 mM) levels of KCl had any effect on *T*_*m*_ (compared with LDH with no additives). The presence of low levels (5 mM) of glucose did not affect the *T*_*m*_ but high levels of glucose (500 mM) significantly increased *T*_*m*_ of both control (76.28±0.09 °C) and dehydrated (76.01±0.02 °C) LDH as compared with their respective values with no additives (*p* < 0.05,*n* = 3 for each). Low levels of urea (20 mM) significantly decreased the *T*_*m*_ of control (75.01±0.03 °C) (*p* < 0.05,*n* = 3), as did higher levels of urea (200 mM) for control (75.04±0.02 °C) (*p* < 0.05,*n* = 3) as compared with their respective values with no additives.

**Figure 8:**
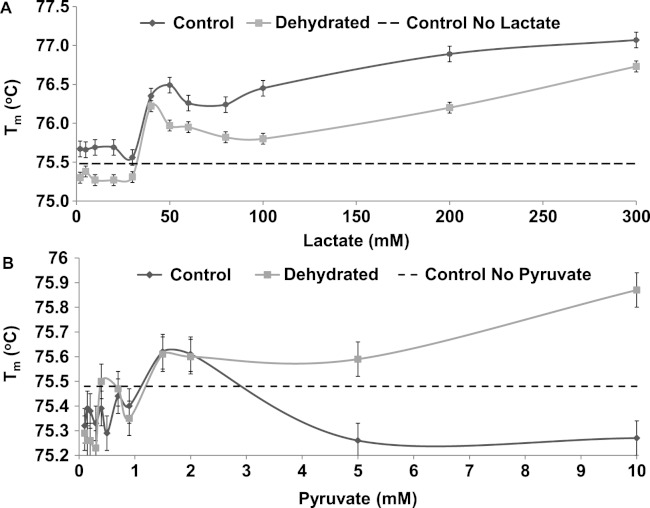
The effect of rising concentrations of lactate and pyruvate on the *T*_*m*_ of purified LDH from muscle of control and dehydrated wood frogs.

The *T*_*m*_ of both control and dehydrated LDH changed similarly in response to changing levels lactate, rising by over 1 °C as lactate increased from 0 to 300 mM (Fig. 8A). However, dehydrated LDH did show a noticeable increase in *T*_*m*_ at higher pyruvate concentrations (5–10 mM) that was not observed with control (Fig. 8B).

### Posttranslational modification of LDH

**Figure 9:**
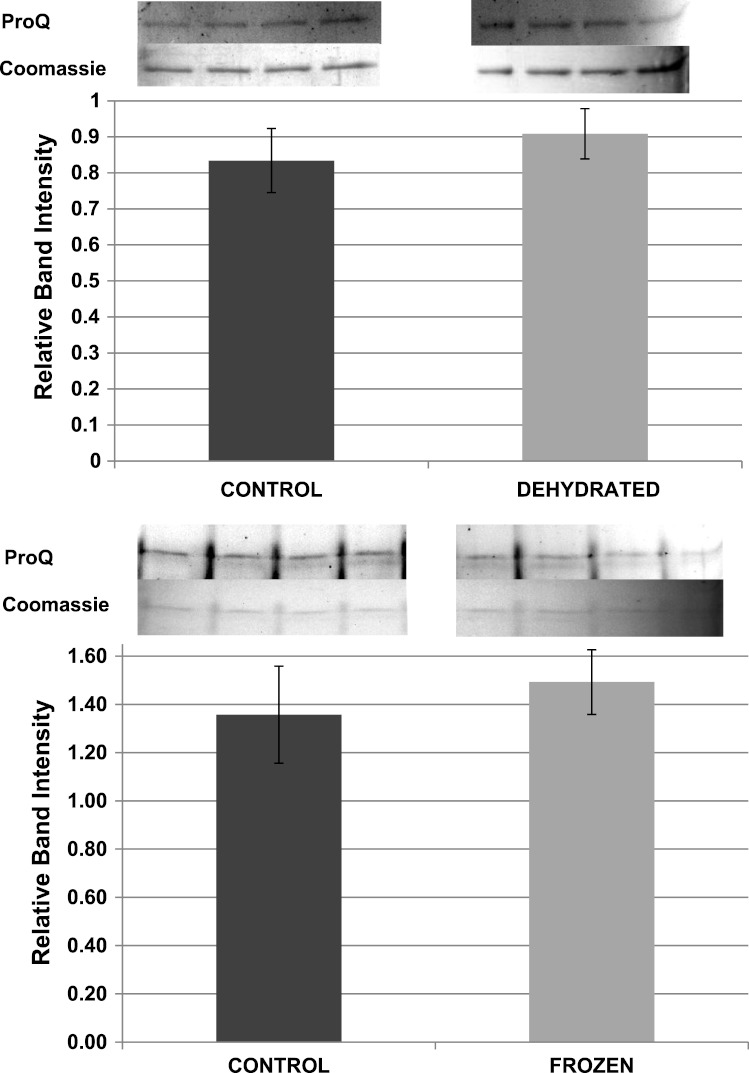
Relative phosphorylation levels of purified muscle LDH from control, frozen and dehydrated frogs. Equal amounts of protein were loaded into each lane of SDS-PAGE gels. ProQ Diamond band density in each lane was normalized against the density of the same band when restained with Coomassie blue. The histogram shows mean ± SEM, *n* = 8 independent determinations. Four representative bands from the ProQ Diamond and Coomassie blue stained gels are shown above each histogram.

Pro-Q Diamond phosphoprotein stain was used to assess the relative phosphorylation state of purified LDH from control, frozen and dehydrated frogs. ProQ band intensities were standardized against the corresponding intensity of the same LDH band after restaining with Coomassie blue. However, standardized Pro-Q fluorescence band intensities showed no significant differences between control, frozen and dehydrated conditions indicating no change in relative protein phosphorylation between the three states (Fig. 9).

**Figure 10:**
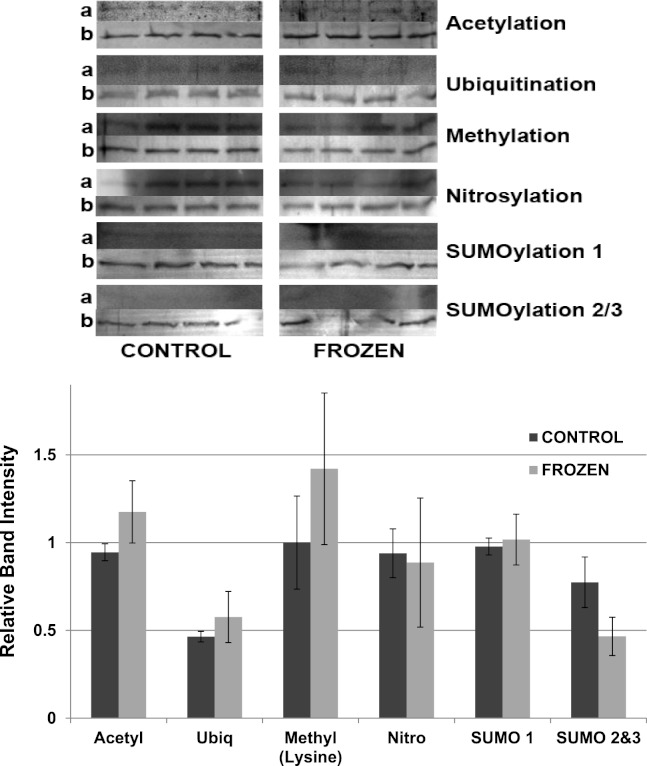
Posttranslational modifications of purified muscle LDH from control and 24 h frozen frogs. Equal amounts of protein were loaded into each lane of SDS-PAGE gels. Immunoblot band density in each lane was normalized against the density of the same band when restained with Coomassie blue. The upper portion of the figure shows representative bands for gels exposed to each type of antibody: anti-acetyl, anti-ubiquitin, anti-methyl, anti-nitrosyl, anti-SUMO 1 and anti-SUMO 2/3. The histogram shows relative band intensities, mean ± SEM, *n* = 4 independent determinations. Images labeled “a” show the immunoblot chemiluminescence; images labeled “b” show the corresponding Coomassie blue stained bands.

**Figure 11:**
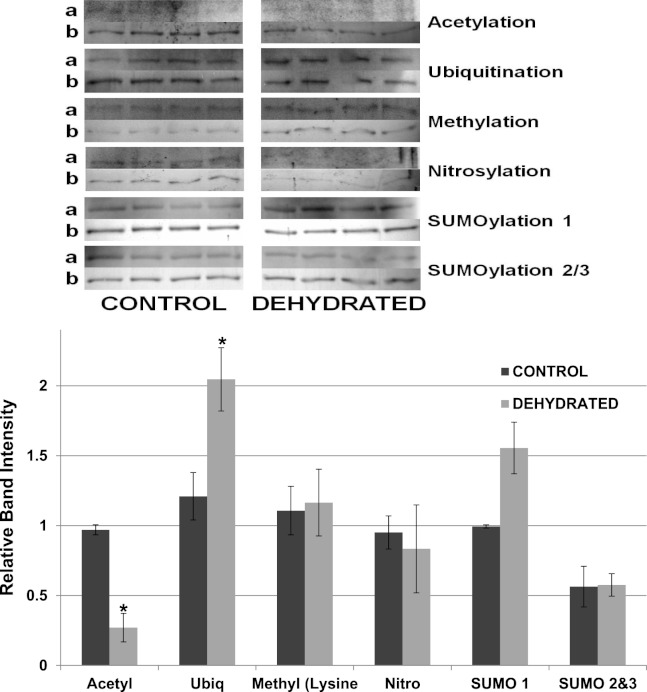
Relative band intensities of Western Blots loaded with samples of 7× concentrated purified muscle LDH from control and 40% dehydrated frogs. Equal amounts of protein were loaded into each lane of SDS-PAGE gels. Immunoblot band density in each lane was normalized against the density of the same band when restained with Coomassie blue. The upper portion of the figure shows representative bands for gels exposed to each antibody (anti-acetyl, anti-ubiquitin, anti-methyl lysine, anti-nitrosyl, anti-SUMO 1 and anti-SUMO 2/3). The histogram shows mean ± SEM, *n* = 4 independent determinations. Images labeled “a” are the western blot chemiluminescence. Images labeled “b” are Coomassie blue stained. * – Significantly different from control LDH by the Student’s t-test (*p* < 0.05).

Other potential posttranslational modifications of wood frog LDH were then assessed by western blotting using purified 7 × concentrated preparations of control and frozen LDH and antibodies that detected different PTMs on proteins. Substantial amounts of methylation and nitrosylation of LDH were detected but other PTMs were only weakly detected: SUMOylation 1, SUMOylation 2&3, acetylation and ubiquitination. However, a comparison of control versus frozen LDH showed no significant differences between the two enzyme forms with respect to any of the PTMs (Fig. 10). When LDH from control versus dehydrated frogs was assessed, the dehydrated enzyme showed a significantly lower level of acetylation (∼75%) and higher level of ubiquitination (∼60%) than the control (*p* < 0.05,*n* = 4 for all) (Fig. 11). A trend towards increased SUMOylation 1 content was also seen but was not significantly different.

### Dot blotting for posttranslational modifications

**Figure 12:**
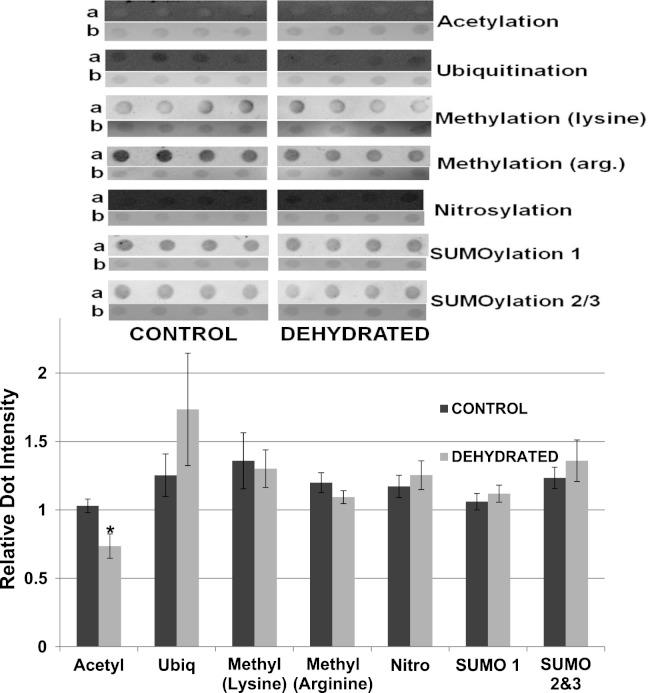
Relative intensities of Dot Blots prepared using samples of 7× concentrated purified muscle LDH from muscle of control and 40% dehydrated frogs. Equal amounts of protein were loaded on each dot. Dot blot intensities were normalized to the corresponding intensity of the Coomassie stained dot after restaining the dot with Coomassie blue. The upper portion of the figure shows representative dot blots exposed to each antibody (anti-acetyl, anti-ubiquitin, anti-methyl lysine, anti-methyl arginine, anti-nitrosyl, anti-SUMO 1 and anti-SUMO 2/3). The histogram shows mean ± SEM, *n* = 4 independent determinations. Images labeled “a” are the dot blot chemiluminescence. Images labeled “b” are Coomassie blue stained. * – Significantly different from control LDH by the Student’s t-test (*p* < 0.05).

To support the results obtained by western blotting, dot blotting was used for an additional assessment of purified control versus dehydrated LDH, using the same antibodies as above, as well as the addition of anti-methyl (arginine). Dot density comparisons supported the results seen for western blots except for ubiquitination (Fig. 12). Dehydrated LDH had a significantly lower level of acetylation (∼30%) by dot blot quantification (*p* < 0.05,*n* = 4 for all). Although a trend indicating increased ubiquitination of dehydrated LDH was seen, the difference was not significant from the control.

## Discussion

Dehydration and freezing can be a causes of death for many animals if they do not use preventative measures to deal with high rates of water loss and/or cell damage due to ice crystals. Wood frogs are known to tolerate up to 50% total body water loss during dehydration or the freezing of 65%–70% of their total body water as extracellular ice (Storey & Storey, 2004). A major response to both freezing and dehydration is the activation of liver glycogenolysis that produces large amounts of glucose for export and elevates organ glucose concentrations by 3- to 313-fold to provide colligative resistance to extreme cell water loss and prevent cell volume from dropping below a critical minimum (Storey, 1987; Storey & Storey, 1984; Churchill & Storey, 1993). Both freezing and dehydration can also lead to oxygen limitation of frog tissues. Freezing of extracellular body fluids (as well as termination of breathing and heart beat in the frozen animal) cuts off oxygen supply to tissues and requires all cells to switch to anaerobic glycolysis for their energy needs with a consequent accumulation of lactate as a glycolytic end product (Storey & Storey, 1984; Storey & Storey, 1986). Progressive dehydration can also cause hypoxic stress because heart and lung functions become more difficult as tissues dehydrate and blood thickens, which makes it more difficult to pump; again, this leads to lactate accumulation (Churchill & Storey, 1993). Historically, LDH has been considered to be a high activity, nonregulatory enzyme that responds simply to the availability of its substrates – producing lactate when oxygen is limiting and high glycolytic flux elevates pyruvate and NADH levels and catabolizing the end product when oxygen availability returns. However, it has become increasingly evident in recent years that there are added layers of complexity in the control and coordination of enzymes within metabolic pathways, even when the enzymes themselves appear to be responding simply to substrate availability. In particular, many types of PTMs of cell proteins/enzymes have been identified but their occurrence and influence on individual enzymes and on the pathways in which they participate is still largely unknown for most enzymes. Our work on animal adaptation to environmental stress has demonstrated the widespread use of reversible protein phosphorylation as both a mechanism of individual enzyme control and of global metabolic rate depression (Storey & Storey, 2007). Our studies to date have mainly focused on known regulatory enzymes in metabolic pathways (e.g. phosphofructokinase and pyruvate kinase within glycolysis) but recently we have become interested in whether other, apparently nonregulatory, enzymes are also influenced by protein phosphorylation and/or are additionally subject to other forms of PTMs. This was our motivation to analyze the properties, regulation and posttranslational modification of wood frog muscle LDH to provide insights into enzyme features that could facilitate LDH function during dehydration and freezing. The present study demonstrates that LDH isolated from skeletal muscle of control and dehydrated wood frogs shows substantially different properties that can be linked with differences in the covalent modification of the enzyme, including protein phosphorylation, acetylation and ubiquitination.

Significant differences in the properties of skeletal muscle LDH were found between the enzyme from control and dehydrated frogs, whereas there were no differences in properties between control and frozen LDH (Table 2). Therefore, most of our subsequent analyses compared muscle LDH from control and dehydrated frogs. Table 2 shows that the *K*_*m*_ lactate and *K*_*m*_ NAD values of dehydrated LDH were both significantly lower than those of control LDH at 22 °C as was the *K*_*m*_ pyruvate of dehydrated LDH lower than control LDH when assayed at 22 °C and 32 °C. In addition, dehydrated LDH was more susceptible to inhibition by urea and glucose at 22 °C (Table 2; Figs. 3 and 4). Urea inhibition of LDH has been reported to arise due to interference by urea with the active site of LDH (Rajagopalan, Handler & Fridovich, 1961) but with *I*_50_ values in the 2–3 M range, urea might be causing generalized protein denaturation instead. Both *K*_*m*_ pyruvate (Table 2) and *K*_*m*_ lactate (Fig. 2) of control LDH increased with increasing assay temperature, rising 2–3 fold between 7° and 32 °C assay temperatures, however, *K*_*m*_ lactate of dehydrated LDH increased instead at 7 °C. The stable differences in kinetic properties listed above for control and dehydrated LDH argue for a stable modification of the muscle enzyme when frogs undergo dehydration. As will be discussed further below, this appears to be the result of a covalent modification of the enzyme protein.

Under conditions of water stress (dehydration on land, hyperosmotic conditions in water), amphibians elevate the concentration of urea in their blood and tissues as a colligative defense against water loss from their bodies. Levels can rise to over 300 mM during seasonal estivation in desert frogs and toads that estivate for many months of the year (Hillman et al., 2009) and levels up to 90 mM have been measured in wood frogs under dry conditions (Costanzo & Lee, 2005). The very high *I*_50_ urea values of 3.16±0.12 M for control LDH (3.10±0.10 M for frozen) and 2.12±0.03 M for dehydrated LDH are such that the level of urea accumulation seen under water stress in wood frogs ( < 100 mM) would be unlikely to influence LDH function in vivo (Table 2). However, high urea is often used to denature proteins and gain information about protein structure and stability. As such the differences between control/frozen and dehydrated LDH suggests a more flexible structure of dehydrated LDH that unfolds more easily in response to urea. Differential sensitivity to urea was also found in an analysis of freezing effects on wood frog hexokinase; *I*_50_ urea values were 5.3 M for control and significantly lower at 4.5 M for frozen hexokinase (Dieni & Storey, 2011). Interestingly, when *I*_50_ urea was assessed after LDH was pre-incubated with urea for 35 min, the *I*_50_ urea of dehydrated LDH did not change further whereas the *I*_50_ of control LDH decreased by more than 50% to a level similar to that of the dehydrated enzyme (Fig. 4). This suggests that control LDH is more resistant to urea and denatured on a slower time course and these data again suggest that there are structural differences between the two enzyme forms.

Wood frogs accumulate high levels of glucose during dehydration and freezing and hence the effects of high glucose on enzyme function were of interest. Figure 2 shows *K*_*m*_ values for lactate in the presence of 400 mM glucose. For assays done at 22 °C, a comparison with the data shown in Table 2 indicates that glucose had no effect on the *K*_*m*_ of control LDH but doubled the value for dehydrated LDH to 11.87±1.19 mM. Glucose at very high levels also had an inhibitory effect on LDH activity (Fig. 3). To our knowledge, glucose has never been reported to inhibit LDH. However, the glucose levels that were inhibitory in our study (2–3.2 M) were well above physiological glucose concentrations in skeletal muscle of frozen or dehydrated frogs (typically in the 20–50 mM range) (Storey & Storey, 1988) and hence, glucose would not have a substantial effect on LDH function in vivo. However, *I*_50_ values for glucose at 22 °C were significantly lower for the dehydrated enzyme than for control LDH. This provides another indication of stability differences between the two enzyme forms as also does the different effects of low versus high temperature change on the *I*_50_ value of the two LDH forms.

The thermal stability of LDH was examined using DSF to measure the mid-point of unfolding (*T*_*m*_) value under different conditions. Figure 6 shows that *T*_*m*_ values for control and dehydrated LDH followed similar patterns with pH change between pH 6–9 although *T*_*m*_ was always lower for the dehydrated enzyme, indicating a less stable form as was also indicated by the *I*_50_ urea data. Data at pH 3 also support this with a large 0.6 °C difference between the two forms. The DSF method also allowed us to test the effects of potential stabilizers or destabilizers on the LDH protein. Figure 7 shows that KCl and low glucose (5 mM is physiological in control frogs) had little effect on protein unfolding. However, high glucose significantly increased the *T*_*m*_ value indicating a protective effect of the sugar against protein unfolding. Sugars and sugar alcohols (e.g. glycerol, sorbitol) typically exhibit stabilizing effects on protein structure at high temperatures and are also well known to prevent freeze denaturation of proteins which accounts for their widespread use as cryoprotectants both in cold-hardy animals and in cryomedicine (Arakawa & Timasheff, 1985; Fink, 1986; Storey et al., 1991). By contrast, urea destabilized control LDH resulting in significantly lower *T*_*m*_ values as compared with enzyme assessed without additives. Interestingly, this effect of urea on high temperature unfolding occurred at low concentrations of urea (20 or 200 mM) as opposed to the *I*_50_ values of 2–3 M seen in Table 2 and Fig. 4. This indicates a cooperative effect of high temperature and urea on protein unfolding. Increasing concentrations of L-lactate above about 50 mM also elevated *T*_*m*_ of LDH in a parallel pattern for the control and dehydrated enzyme forms (Fig. 8). Indeed, the protective effect of lactate at 200–300 mM on *T*_*m*_ was as good as or better than the effect of 500 mM glucose. This might prove to be a common effect of lactate since lactate was just as effective as glycerol or glucose in the freezing protection of insect glucose-6-phosphate dehydrogenase with 20–25 mM providing half-maximal protection of enzyme activity after 1 h freezing at −77 °C (Storey et al., 1991). Thus, although lactate does not rise to high levels in frogs during freezing or dehydration in vivo (typically 15–20 mM) (Storey & Storey, 1986; Churchill & Storey, 1993), its gradual accumulation over time will add to the total carbohydrate osmolyte pool within cells and contribute to the defense of macromolecules against denaturation when environmental stress is prolonged. Concentrations of pyruvate of about 2 mM improved the *T*_*m*_ value of both LDH forms but higher levels had differential effects; 5–10 mM pyruvate further improved stability of dehydrated LDH but destabilized the control enzyme. This provides another indication of structural difference between the control and dehydrated forms of LDH.

The data discussed above show many instances of significant differences in properties and stability between LDH isolated from skeletal muscle of control and dehydrated wood frogs; parameters affected included substrate *K*_*m*_ values for NAD, lactate and pyruvate, *I*_50_ urea, effect of temperature on *K*_*m*_ lactate, and *T*_*m*_ values for protein unfolding. All of these data suggest a stable structural difference between the control and dehydrated LDH forms, most probably provided by a PTM. We first investigated protein phosphorylation since it is widely used by animals to create stress-responsive changes in enzyme properties (Storey & Storey, 2007). However, Pro-Q Diamond phosphoprotein staining of LDH showed no differences in the bound phosphate content of skeletal muscle LDH from control, frozen or dehydrated frogs (Fig. 9). We next evaluated a range of other PTMs using immunoblotting to detect differences in acetylation, ubiquitination, methylation, nitrosylation, and SUMOylation of the LDH protein. A comparison of the enzyme from control and frozen frogs found no differences in any of these PTMs (Fig. 10) but the comparison of LDH from control and dehydrated frogs showed significant differences in protein acetylation and ubiquitination (Fig. 11). The difference in protein acetylation was also apparent when a dot blot method of analysis was used (Fig. 12). In both cases, dehydrated LDH showed significantly reduced protein acetylation as compared with the control enzyme as well as significant differences in enzymatic properties. Among amphibians, the only previous report of protein acetylation involvement in metabolic regulation was the demonstration of acetylation of the α-chain N-terminus of hemoglobin in the Andean frog, *Telmatobius peruvianus*, that increased oxygen affinity of the blood protein as compared with lowland frogs (Weber et al., 2002). However, we recently reported the first instance of differential acetylation of an enzyme of intermediary metabolism in response to an environmental stress (oxygen deprivation) in the anoxia-tolerant turtle, *Trachemys scripta elegans*. An analysis of LDH from turtle liver showed a 70% higher content of acetylated lysine residues on the anoxic enzyme form versus aerobic LDH (Xiong & Storey, 2012). This was correlated with significant differences between the two enzyme forms in *V*_max_, *K*_*m*_ pyruvate, *I*_50_ pyruvate and *T*_*m*_. Ubiquitination is well-known to mark proteins for degradation and differences in the pattern of ubiquitination determines whether individual proteins are brought to autophagosomes or to proteasomes for degradation (Benbrook & Long, 2012). The ubiquitin proteasome system is an integral part of the stress response in animals and has been particularly well studied in hypoxia and heat shock (Flick & Kaiser, 2012). In particular, the interplay between ubiquitin ligases and stress-responsive transcription factors has been well-studied and is crucial to the up-regulation of various stress-responsive genes (Flick & Kaiser, 2012). Ubiquitination also functions to target damaged or unfolded proteins for degradation under stress conditions. The current data on LDH ubiquitination levels in control versus dehydrated states, although not consistent between Figs. 11 and 12, is suggestive of enhanced ubiquitination of LDH in the stress condition. More work will be needed to confirm differential ubiquitination of the enzyme, explore the regulatory mechanisms involved, and understand the physiological relevance of the response.

On the other hand, the evidence for differential acetylation of LDH in control versus dehydrated states is strong (Figs. 11 and 12). Acetylation is the enzyme-catalyzed addition of an acetyl group (C_2_H_3_O) to specific amino acid residues, typically lysine. Early studies reported that acetylation affected the kinetics of carboxypeptidase A, acetylation within the active site causing inhibition of activity (Whitaker et al., 1965). Very recently, however, lysine acetylation of metabolic enzymes has been recognized as a widespread and important PTM in metabolic control in both normal and disease states (Xiong & Guan, 2012). In human liver, acetylation of enzymes was found to change in response to metabolic fuels including glucose, amino acids and fatty acids (Xiong et al., 2010). Almost all enzymes in glycolysis, gluconeogenesis, the tricarboxylic acid (TCA) cycle, urea cycle, fatty acid metabolism, and glycogen metabolism showed covalently modified lysine groups and metabolic consequences included activation of malate dehydrogenase of the TCA cycle, inhibition of argininosuccinate lyase in the urea cycle, and destabilization of phosphoenolpyruvate carboxykinase in gluconeogenesis (Xiong et al., 2010). New studies have also reported acetylation-mediated regulation of the M2 isoform of pyruvate kinase in tumor cells (Lv et al., 2011). Lysine acetylation also negatively regulated liver glycogen phosphorylase activity both directly and by promoting dephosphorylation by protein phosphatase 1 (Zhang et al., 2012). The lysine locking activity of acetylation interferes with other potential lysine modifications (e.g. methylation, ubiquitination, SUMOylation, neddylation, biotinylation) (Yang & Seto, 2008) thereby implicating a regulatory role for protein acetylation in competition with other PTMs.

## Conclusion

The present data show that wood frog muscle LDH exhibits stable changes in its enzymatic properties in response to cellular dehydration. A concurrent reduction in protein acetylation implicates this PTM as the molecular mechanism underlying dehydration-responsive LDH modification. The low acetyl dehydrated form of LDH showed higher affinity for substrates, but lower tolerance for urea inhibition and lower thermal stability as compared to control LDH. The present study of frog muscle LDH together with our recent work on turtle LDH (Xiong & Storey, 2012) suggest that differential acetylation of metabolic enzymes will soon prove to have widespread roles in the adaptive regulation of enzymes to environmental stress.
